# MRI features to aid the identification of lateral temporal bone cephaloceles

**DOI:** 10.1259/bjr.20230014

**Published:** 2023-09-03

**Authors:** Rohit Srinivasan, Rupert J Obholzer, Steve EJ Connor

**Affiliations:** 1 Department of Radiology, Guy’s Hospital, London, United Kingdom; 2 Department of Otolaryngology, Guy’s and St. Thomas’ Hospitals NHS Foundation Trust, London, United Kingdom; 3 School of Biomedical Engineering and Imaging Sciences, St Thomas’ Hospital, King’s College London, London, United Kingdom; 4 Department of Neuroradiology, King’s College Hospital, London, United Kingdom

## Abstract

**Objectives:**

To evaluate ancillary MRI features which may aid the identification of lateral temporal bone cephaloceles (LTBCs).

**Methods:**

A retrospective cohort study analysed patients with MRI evidence of surgically confirmed spontaneous LTBCs as defined by intracranial contents traversing the tegmen tympani or mastoideum. Cases were identified from radiology and surgical databases. Two observers analysed three-dimensional *T*_2_W temporal bone and whole brain imaging according to *a priori* criteria by consensus, with emphasis on the relationship of any adjacent cerebrospinal fluid (CSF) cleft to the defect. The contents, location, and clinical features of the LTBCs were recorded.

**Results:**

Eighteen patients (11 female, 7 male; mean age 59.3 years, age range 42–86 years) with 20 surgically confirmed spontaneous LTBCs (2 bilateral;16 unilateral) were evaluated. A temporal lobe sulcus or other CSF cleft extending to or traversing the defect was identified in 19/20 (95%) cases. Isointense CSF tympanomastoid signal was present in 41.2% cases, whilst superior semi-circular canal dehiscence was found in 40% of cephaloceles. At least two MRI features of idiopathic intracranial hypertension were seen in 38.9% patients. Cephaloceles were most commonly centred on the tegmen tympani (55%). Meningoencephaloceles were present in 95% cases.

**Conclusion:**

A temporal lobe sulcus or CSF cleft extending to or traversing the defect may aid the identification of LTBCs. Isointense CSF tympanomastoid signal, superior semi-circular canal dehiscence and MRI features of idiopathic intracranial hypertension are only present in under half of LTBCs.

**Advances in knowledge:**

The study details novel ancillary MRI features of LTBCs which may aid their identification.

## Introduction

Spontaneous lateral temporal bone cephaloceles (LTBCs) are characterised by the abnormal passage of intracranial contents into the middle ear and mastoid in the absence of trauma, surgery, tumours, or developmental defects.^1–3^ Although thought to be fairly uncommon, there are increasing reports of spontaneous temporal bone cephaloceles in recent international literature with a prevalence of up to 2% in healthy controls.^1,4–7^

Spontaneous LTBCs are thought to manifest from a multifactorial process involving increased intracranial pressure in conjunction with an anatomical structural predisposition at the site of pneumatisation.^3,8^ The condition has been frequently associated with idiopathic intracranial hypertension (IIH).^9,10^

Diagnosis of the condition is often difficult due to the non-specific constellation of presenting signs and symptoms. Commonly reported symptoms include hearing loss and cerebrospinal fluid (CSF) otorrhea, whilst rarer clinical presentations include seizures.^2,11,12^ Patients are at risk of potentially life-threatening infections such as meningitis, so timely identification is required.^13^

Imaging has an important role in the prompt and accurate diagnosis of the condition and to prevent further complications. Whilst CT can identify temporal bone defects and soft tissue opacification, MRI is optimal for directly demonstrating a LTBC. Thin section spin echo or gradient recalled echo (GRE) *T*_2_W sequences are the mainstay for evaluation. However, detecting the herniation of intracranial contents may be challenging, particularly in the absence of a meningocele. It is therefore important to evaluate ancillary imaging features which aid the identification. In this regard, the configuration of adjacent sulcal spaces and the presence of tympanomastoid fluid may provide indirect evidence of a cephalocele,^3,14,15^ whilst superior semi-circular canal dehiscence (SSCD) and features of IIH are recognised associations.^3,16–18^ Recent studies have described certain clinical and radiological findings in spontaneous LTBCs but have not focussed specifically on the MRI features of this condition.^1,19^

The primary objective of the study was to determine ancillary MRI findings which aid the identification of surgically confirmed spontaneous LTBCs whilst the secondary objectives were to define their contents, location and clinical features.

## Methods and materials

### Patient population

**Figure 1.**
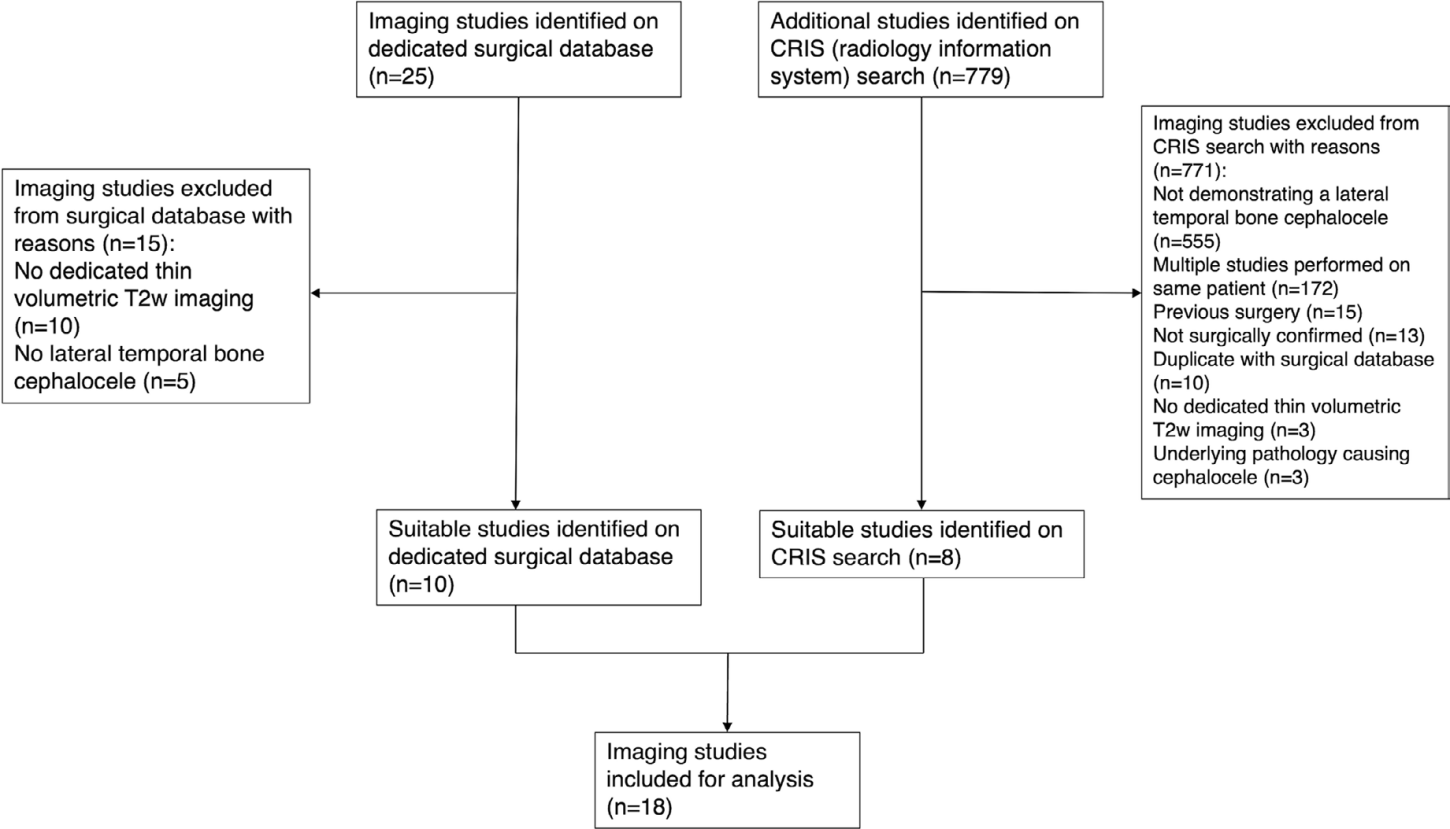
Flow chart summarising the patient selection process.

Institutional approval (project number 13453) to conduct a service improvement project was obtained and patient consent was waived. This was a retrospective cohort study undertaken in a tertiary referral centre. Searches of a radiology imaging system and a dedicated surgical database were conducted from 01/01/2006 to 09/02/2022. The Boolean search comprised the keywords ‘cephalocele’ AND ‘petrous’ OR ‘temporal’ OR ‘ear’ OR ‘mastoid’. Exclusion criteria were cephaloceles secondary to trauma, surgery, tumours or developmental defects, cephaloceles at the petrous apex, previous tympano-mastoid surgery, those not confirmed surgically and those without three-dimensional (3D) *T*_2_W imaging of the temporal bones. The study flowchart shown in Figure 1 summarises the patient selection process.

During this period, 25 MRI studies were found from a dedicated surgical database and supplemented with 779 studies on an additional imaging search. Following this, 786 studies were excluded and MRI studies of 18 individual patients were included for analysis. Demographic data including age, gender and surgical history were collated.

### Clinical and surgical data

Clinical features including presenting signs and symptoms were collected. A diagnosis of IIH was confirmed according to clinical documentation of the patient meeting the Modified Dandy Criteria.^20,21^ Surgical data including the operation date, site, surgical approach and type of cephalocele were collated.

### MRI protocols and image analysis

Axial whole brain *T*_2_W imaging and 3D *T*_2_W dedicated axial imaging of the temporal bones was performed on 1.5 Tesla (T) or 3T MRI systems. With respect to the 3D *T*_2_W axial imaging, CISS (constructive interference in steady state) was performed on 1.5T MAGNETOM Aera or Avanto scanners (Siemens Healthcare; Erlangen, Germany) in 16/20 (80%) cases, with one case (5%) performed on a 3T MAGNETOM Skyra scanner (Siemens Healthcare; Erlangen, Germany), whilst 3D *T*_2_W SPACE (sampling perfection with application optimised contrasts using different flip angle evaluation) was performed on 3T MAGNETOM Skyra scanners (Siemens Healthcare; Erlangen, Germany) in 3/20 (15%) cases. Whilst there was some variation between MRI scanners, the most frequently applied parameters were repetition time 5.8 ms, echo time 2.6 ms, one signal average, FOV 180 x 180 mm, slice thickness 0.7 mm, flip angle 66^0^, acquisition matrix 320 × 320, 420 pixel bandwidth Hz/pixel for the CISS sequence and repetition time 1400 ms, echo time 155 ms, two signal averages, FOV 150 x 150 mm, slice thickness 0.7 mm, flip angle 120^0^, acquisition matrix 320 × 320, 290 pixel bandwidth Hz/pixel for the SPACE sequence.

Two head and neck radiologists with 30 years (SC) and 5 years (RS) experience analysed the MRI studies with the aid of multiplanar reconstructions and according to a priori criteria by consensus.

**Figure 2.**
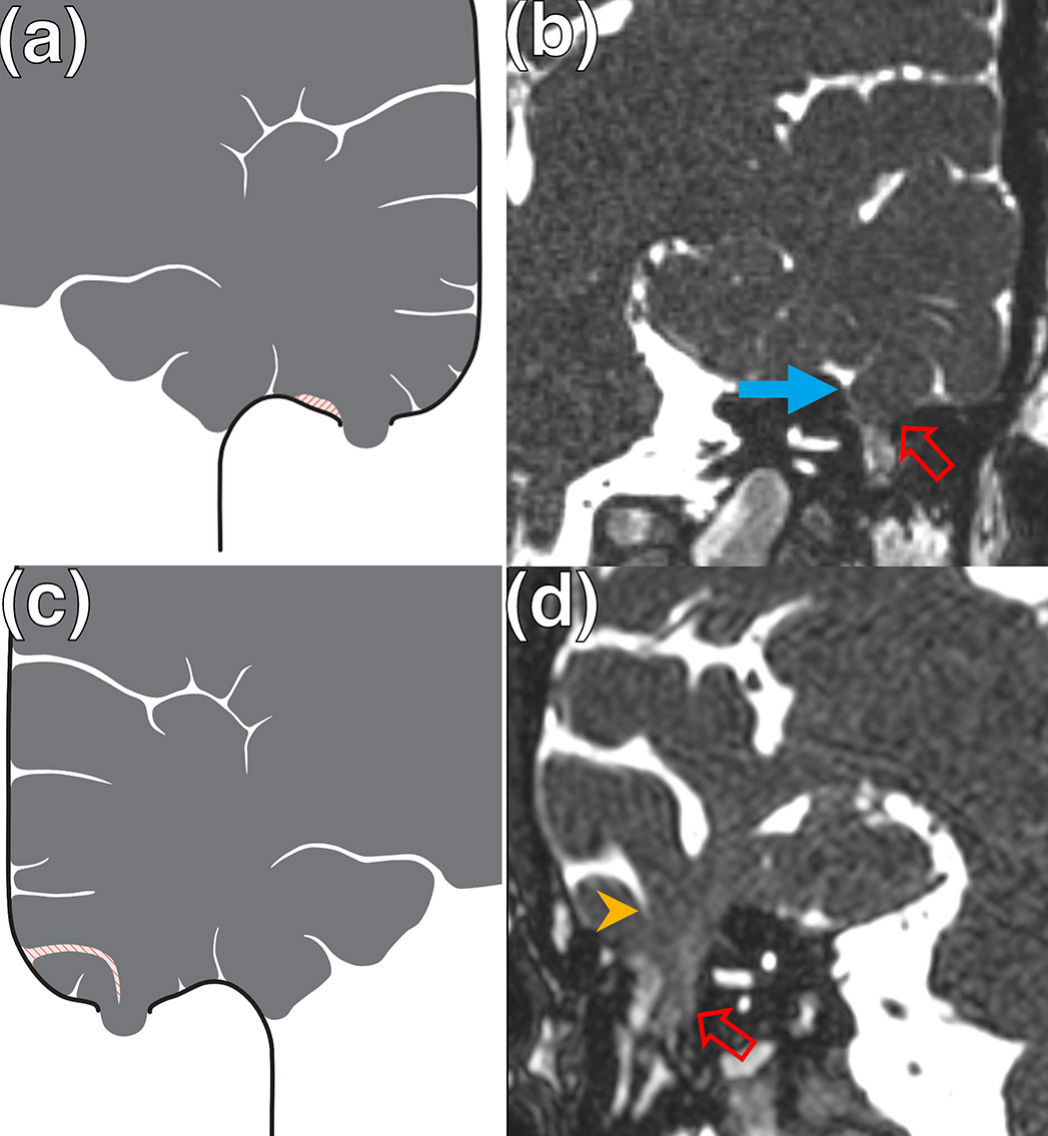
Lateral temporal meningocephaloceles with an adjacent temporal lobe sulcus or other CSF cleft extending to the margin of the defect. (**a, c**) Schematic diagrams depicting lateral temporal bone meningoencephaloceles involving an adjacent temporal lobe sulcus (striped) (**a**)extending to the margin of the defect or other CSF cleft overlying the temporal lobe sulcus (**c**) extending to the centre of the defect (interruption in solid line). (**b, d**) Coronal 3D CISS imaging of the temporal bones demonstrating examples of lateral temporal bone meningoencephaloceles (open arrows) involving either an adjacent temporal lobe sulcus (**b**) extending to the margin of the defect (arrow) or other CSF cleft overlying the temporal lobe sulcus (**d**) extending to the centre of the defect (arrowhead). 3D, three-dimensional; CISS, constructive interference in steady state; CSF, cerebrospinal fluid.

**Figure 3.**
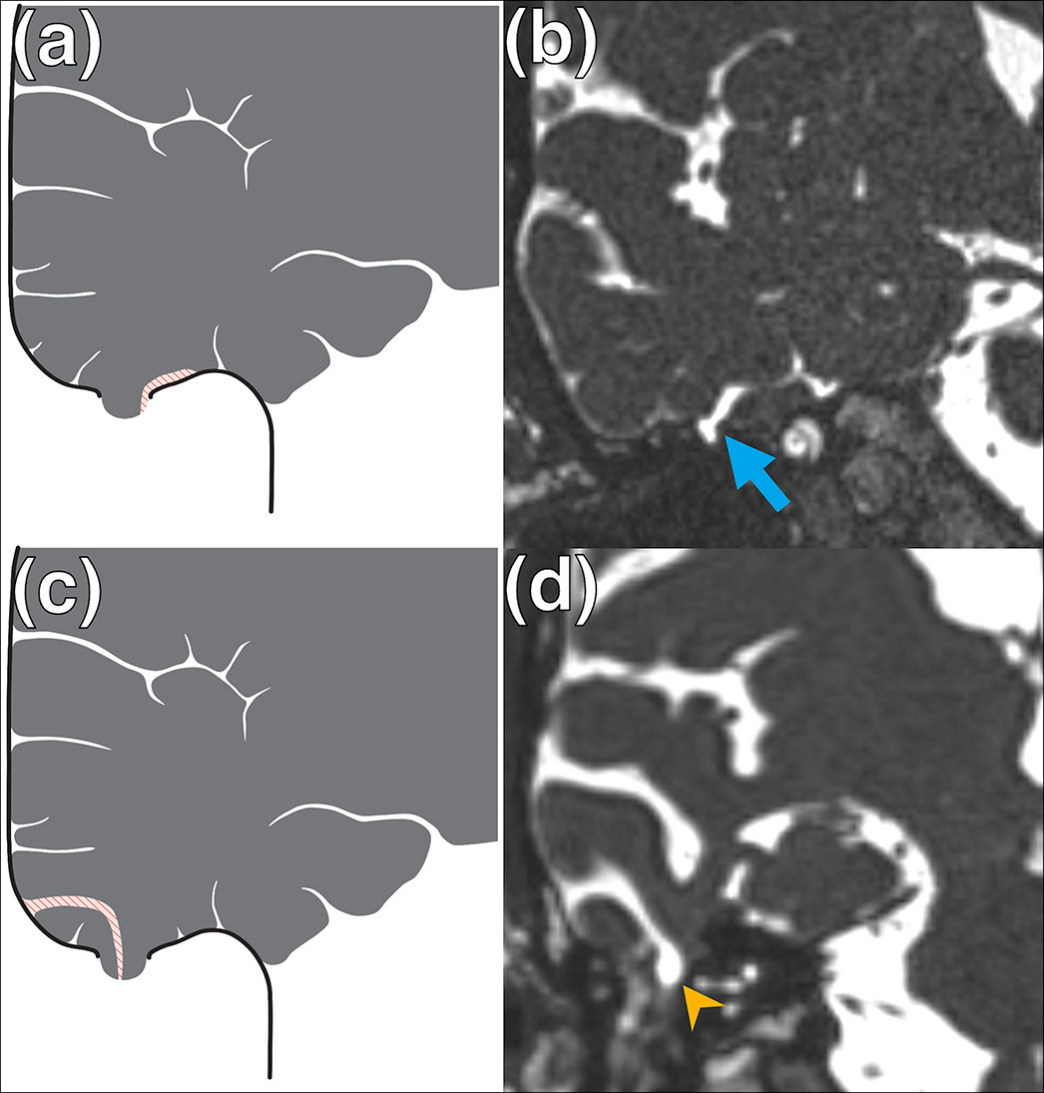
Lateral temporal bone meningoencephaloceles with an adjacent temporal lobe sulcus or other CSF cleft traversing the defect. (**a, c**) Schematic diagrams depicting lateral temporal bone meningoencephaloceles depicting an adjacent temporal lobe sulcus (striped) traversing the margin of the defect (**a**) or other CSF cleft overlying the temporal lobe sulcus (**c**) traversing the centre of the defect (interruption in solid line). (**b, d**) Coronal 3D CISS imaging of the temporal bones demonstrating examples of right sided lateral temporal bone meningoencephaloeceles involving either a traversing adjacent temporal lobe sulcus (**b**) along the margin of the defect (arrow) or other CSF cleft overlying the temporal lobe sulcus (**d**) through the centre of the defect (arrowhead). 3D, three-dimensional; CISS, constructive interference in steady state; CSF, cerebrospinal fluid.

**Figure 4.**
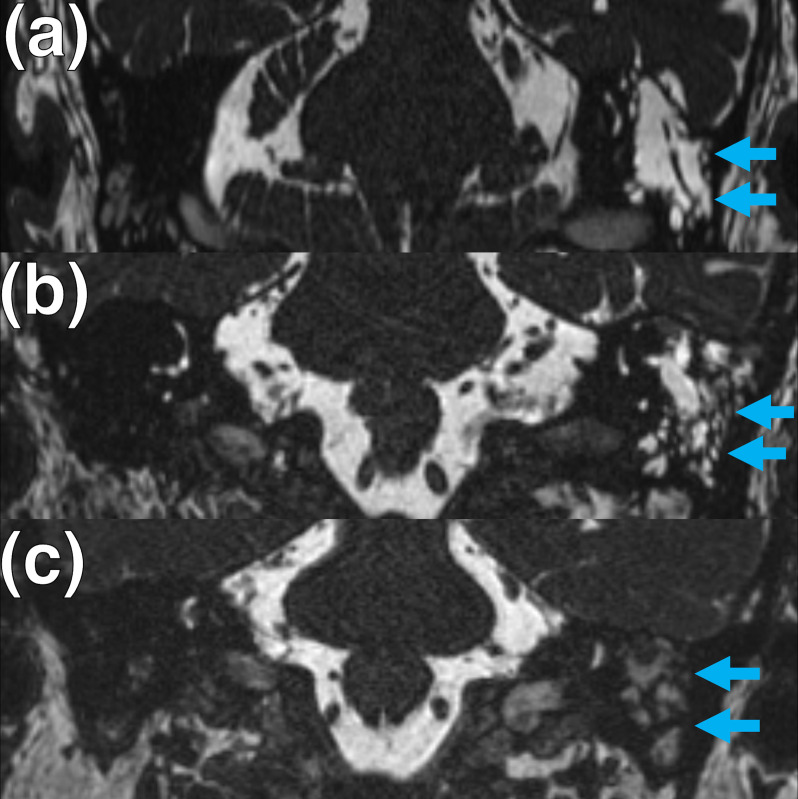
Tympanomastoid signal abnormality in patients with lateral temporal bone cephaloceles. (**a**, **b, c**) Coronal 3D CISS imaging of the temporal bones demonstrating examples of tympanomastoid signal abnormality (arrows) relative to the surrounding CSF in patients with meningoencephaloceles. Examples of (**a**) entirely isointense, (**b**) partially isointense and (**c**) entirely different tympanomastoid signal relative to CSF are demonstrated. 3D, three-dimensional; CISS, constructive interference in steady state; CSF, cerebrospinal fluid.

MRI ancillary features and associations of LTBCs were evaluated. Firstly, the presence of either an adjacent temporal lobe sulcus or another CSF cleft overlying the temporal lobe was recorded if it extended to (Figure 2) or traversed (Figure 3) the defect. Such a CSF cleft may extend to or traverse the margin of the defect (Figures 2a and 3a) or alternatively may extend to or traverse the centre of the defect (Figures 2c and 3c). The presence of adjacent sulcal widening or asymmetric enlargement of the adjacent intracranial subarachnoid space was also recorded. Secondly, in cases with 3D CISS sequences available, the tympanomastoid signal was compared to that of CSF (CSF isointense, partially CSF isointense or completely different relative to CSF signal) (Figure 4). Thirdly, the presence of SSCD was evaluated on oblique coronal and sagittal 3D multiplanar reformats. Fourthly, the presence of **≥**2 MRI features of IIH was recorded (Table 1).^18,21^ Finally, the MRI features of the cephalocele were recorded with respect to the contents (meningocele or meningoencephalocele) and location (tegmen tympani, medial or lateral tegmen mastoideum, alone or in combination).

**Table 1.** Lists the specific MRI features of IIH that were investigated

Criterion	MRI feature of IIH
(i)	‘Empty’ sella appearance
(ii)	Osteodural defects or meningoceles elsewhere
(iii)	Posterior globe flattening
(iv)	Optic nerve sheath distension
(v)	Optic nerve tortuosity

IIH, idiopathic intracranial hypertension.

If at least two of the features were demonstrated, this was recorded as positive for IIH.^18,21^

### Statistical analysis

Descriptive statistics were applied using Microsoft^®^ Excel for Mac 2021.

## Results

### Demographics

18 patients (11 female, 7 male; mean age 59.3 years, age range 42–86 years) with surgically confirmed spontaneous LTBCs were identified during the study period. Two patients had bilateral defects and the final cohort comprised 20 spontaneous LTBCs.

### Ancillary MRI features

**Figure 5.**
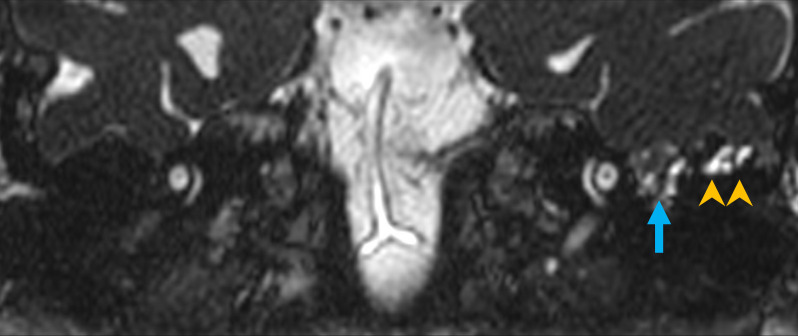
Coronal 3D CISS imaging demonstrates a left sided lateral temporal bone meningoencephalocele (arrow) centred upon the tegmen tympani. There is no associated CSF signal cleft extending to or traversing the defect, nor associated adjacent asymmetric sulcal widening. Small volume tympanomastoid signal abnormality (arrowheads) isointense relative to CSF is demonstrated. 3D, three-dimensional; CISS, constructive interference in steady state; CSF, cerebrospinal fluid.

A high *T*_2_W CSF signal cleft traversing the margin or centre of the defect was identified in 12/20 (60%) LTBCs. A high *T*_2_W CSF signal cleft, extending to the margin or central portion of the defect was detectable in 7/20 (35%) cases. Overall, a CSF signal cleft which either extended to or traversed the defect was found in 19/20 (95%) LTBCs (Figures 2 and 3). The single LTBC without an associated CSF signal cleft extending to or traversing the defect is seen in Figure 5. Adjacent sulcal widening or asymmetric enlargement of the adjacent intracranial subarachnoid space was observed in 17/20 (85%) LTBCs.

There was *T*_2_W tympanomastoid signal abnormality demonstrated in all cases. In cases where 3D CISS imaging was performed, the tympanomastoid signal change was entirely CSF isointense in 7/17 (41.2%), partially CSF isointense in 7/17 (41.2%) or different to CSF signal in 3/17 (17.6%) cases.

**Figure 6.**
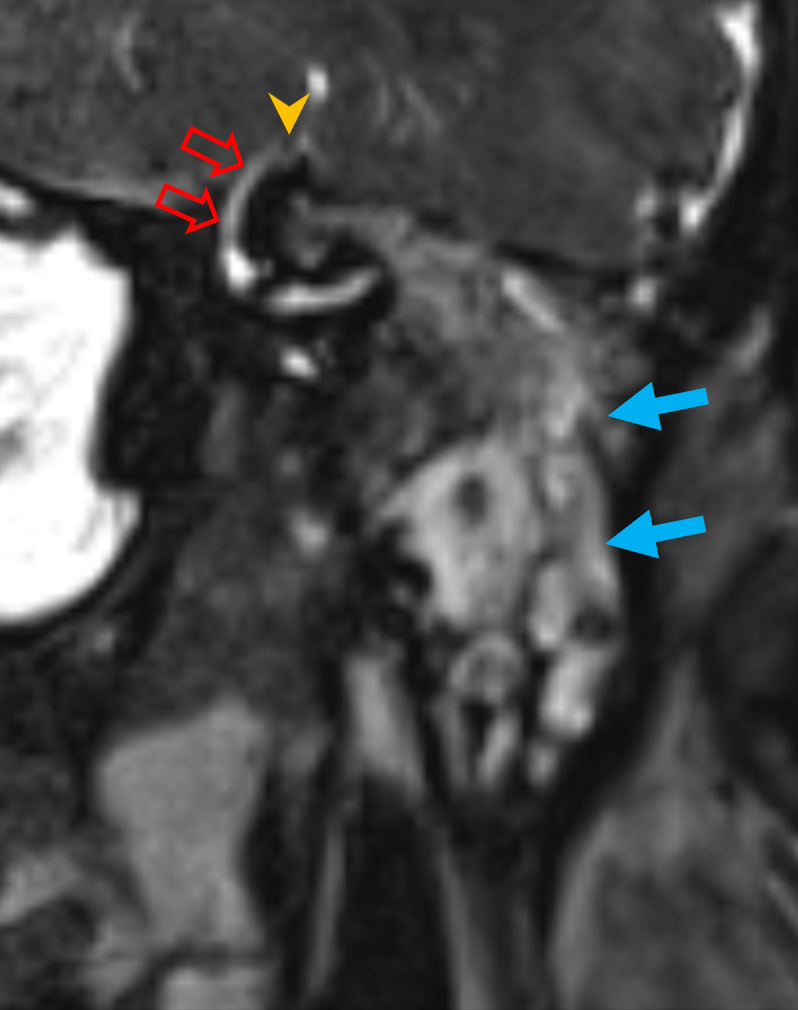
SSCD associated with a lateral temporal bone meningoencephalocele. Coronal oblique 3D CISS imaging demonstrates a left-sided SSCD (arrowhead; open arrows—intact superior semicircular canal) in a patient with a spontaneous lateral temporal bone meningoencephalocele and associated tympanomastoid signal abnormality (filled arrows) that is of different signal relative to CSF. 3D, three-dimensional; CISS, constructive interference in steady state; CSF, cerebrospinal fluid; SSCD, semi-circular canal dehiscence.

Eight of 20 (40%) cases in the cohort were found to demonstrate SSCD on MRI (Figure 6).

At least two MRI imaging features of IIH were demonstrated in 7/18 (38.9%) patients. There was one patient (14.3%) with IIH imaging features without a corresponding clinical diagnosis. In total, nine (50%) patients were found to have clinically confirmed IIH.

Table 2 summarises the ancillary MRI features of spontaneous LTBCS in our cohort of cases.

**Table 2.** Summarising the ancillary MRI features of spontaneous LTBCS in our cohort of cases

	Values, n (%)
High *T*_2_W CSF signal temporal lobe sulcus	
Traversing defect	12/20 (60)
At the margin of defect	7/20 (35)
None	1/20 (5)
Mastoid high *T*_2_W signal on 3D CISS imaging (relative to CSF)^*a*^	
Isointense	7/17 (41.2)
Partially isointense	7/17 (41.2)
Different	3/17 (17.6)
None	0/17
Imaging features of intracranial hypertension^*b*^	
Yes	7/18 (38.9)
No	11/18 (61.1)
Superior semi-circular canal dehiscence	
Yes	8/20 (40)
No	12/20 (60)

3D, three-dimensional; CISS, constructive interference in steady state; CSF, cerebrospinal fluid; LTBCS, lateral temporal bone cephaloceles.

a 3* 3D CISS imaging availble in 17/20 cases.

b ≥ 2† ≥2 MRI imaging features^18,21^ ; *n* = 18 patients.

### Content and location of cephaloceles

**Figure 7.**
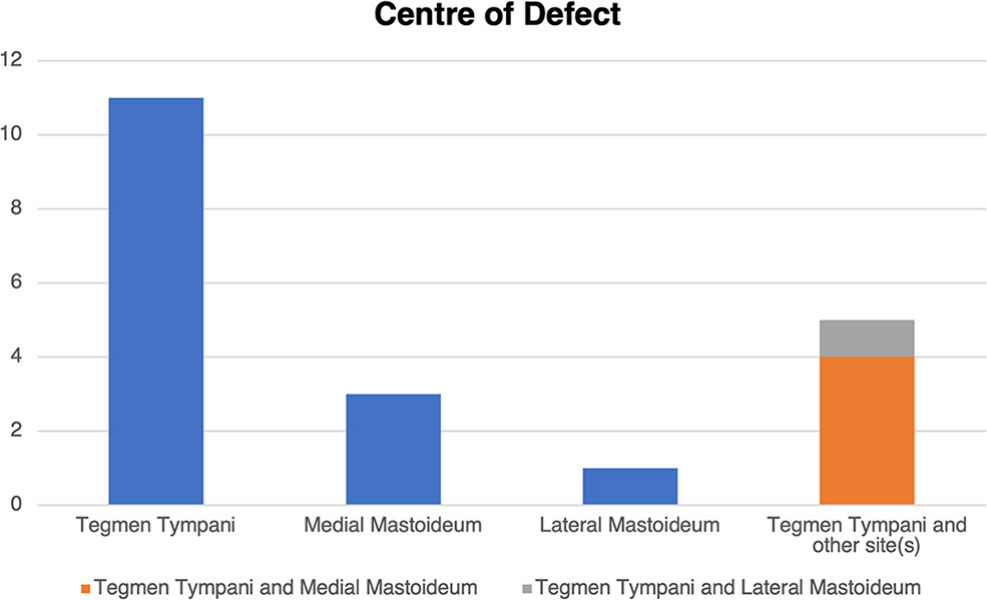
Bar chart demonstrating the anatomical distribution of the centre of the defect.

MRI demonstrated meningoencephaloceles in 19/20 (95%) cases. Cephaloceles were most frequently centred upon the tegmen tympani, either alone in 11/20 (55%) LTBCs or in combination with other sites in 16/20 (80%) of cases (Figure 7).

### Clinical and surgical features

Hearing loss was the most common presenting symptom, experienced by 16/18 (88.9%) patients. Seven (38.9%) patients presented with otorrhea, whilst three (16.7%) patients had meningitis. No patient presented with a seizure. The 20 cases were surgically confirmed using transmastoid (12/20), middle fossa (7/20) or combined (1/20) approach repairs. Two (11.1%) patients underwent bilateral surgical repairs.

## Discussion

A high *T*_2_W CSF signal cleft was identified to extend to or traverse the bony defect in almost all (95%) surgically confirmed LTBCs on MRI and represents a key MRI feature which may aid their identification. Adjacent LTBC sulcal widening or asymmetric enlargement of an intracranial subarachnoid space was also seen in 85% of cases. Minor ancillary features such as tympanomastoid signal entirely isointense to CSF, SSCD and MRI features of IIH are only present in under half of LTBCs and their absence should therefore not deter the diagnosis.

The current radiological literature characterising the imaging features of spontaneous LTBCs is limited. Two recent studies investigating a cohort of spontaneous LTBCs predominantly focussed upon the clinical features and CT imaging findings.^1,19^ Our study adds to this literature by evaluating associated MRI features in a cohort of surgically confirmed cases which may aid their detection. Previous studies investigating the MRI appearances of cephaloceles more generally have included descriptions regarding the associated traction of adjacent sulci towards a cephalocele. For example, this may manifest as an accompanying herniation of a pouch of CSF or asymmetric enlargement of the adjacent intracranial subarachnoid space.^3,14^ However, our study expands on this by specifically detailing the prevalence and description of a *T*_2_W CSF signal cleft extending to or traversing the defect in spontaneous LTBCs.

Additional ancillary MRI features were chosen for evaluation on the basis of known associations. Firstly, unilateral tympanomastoid effusion is known to be associated with LTBCs, both clinically and on imaging studies.^15,22^ GRE 3D *T*_2_W sequences such as CISS are optimal for the analysis of the differing fluid composition of tympanomastoid fluid due to the utilisation of heavier T2 weighting and increased signal-to-noise ratios.^23,24^ Breen et al studied patients with temporal bone CSF leaks, although without cephaloceles in the majority of cases, and found that tympanomastoid signal isointense to CSF was 100% specific and 76% sensitive for the presence of a CSF leak.^24^ Interestingly, only 41.2% of our LTBCs demonstrated entirely CSF isointense tympanomastoid fluid. This discrepancy may relate to the partial or transient obstruction of the defect, in the presence of a cephalocele, resulting in an increased protein content of any tympanomastoid CSF leak.^25^ Secondly, SSCD and spontaneous LTBCs are both linked to thinning of the tegmen tympani and mastoideum.^16,17^ The high rate of SSCD (40%) in our cohort was similar to a recent study evaluating the prevalence in patients with lateral temporal bone cephaloceles,^26^ and much higher than the estimated prevalence of 0.7–4.0% in asymptomatic individuals.^27,28^ Whilst SSCD was absent in the majority of cases, the finding is of potential clinical importance since concomitant SSCD and LTBC repair can be performed using either a transmastoid or middle fossa approach.^29,30^ Finally, there is a known relationship between skull base cephaloceles and IIH.^9,10,31^ In our cohort, 38.9% of patients had MRI features of IIH, whilst half had a clinically confirmed diagnosis. This is higher than previously described in a retrospective case–control study investigating the rate of spontaneous intracranial meningoceles in IIH patients.^9^

MRI can also have an important role in delineating the intracranial contents within a cephalocele. Meningoencephaloceles accounted for the majority of cephaloceles in the study. No previous studies have delineated the type of cephalocele using MRI in a cohort of patients with spontaneous LTBCs. The finding is of particular importance since the type of cephalocele has implications on pre-operative planning and outcome.^32^ In addition, identifying the centre of the defect is another important factor in influencing surgical approach.^22^ The tegmen tympani was involved as the centre of defect, either alone or in conjunction with other sites, in the majority (80%) of LTBCs in our cohort. The rate of tegmen tympani involvement in our study is towards the higher end compared to recent studies.^1,19^ The non-specific constellation of clinical presenting features in patients with spontaneous LTBCs presents a diagnostic challenge. Hearing loss was the most common symptom and in line with the rate in other recent studies.^1,19^ CSF otorrhea and meningitis are recognised clinical presentations and featured in our cohort, whereas seizures did not.^1,19,33,34^

The authors appreciate there are some limitations to the methodology. Firstly, inherent to the nature of the retrospective study, there were some data points which were not possible to complete. For example, spontaneous temporal bone cephaloceles have been significantly associated with obesity.^35^ However, the body mass indices in most patients were not available. Secondly, the scoring of MRI features was performed by consensus rather than by multiple observers. Whilst *a priori* criteria were applied, the subjective nature of these observations would have benefited from independent scoring and calculation of agreement statistics. Thirdly, our proposed ancillary features have not been fully evaluated for their diagnostic utility since they were not applied to a control group. Finally, the low number of patients in our study somewhat limits the overall generalisability of the results.

## Conclusion

In conclusion, a *T*_2_W CSF signal cleft extends to or traverses the temporal bone defect in almost all cases of surgically confirmed spontaneous LTBCs on MRI, whilst entirely CSF isointense tympanomastoid signal, SSCD and MRI features of IIH are only present in under half of LTBCs. Interrogating the MRI for CSF clefts at the interface of the temporal lobe and temporal bone tegmen may aid the detection of LTBCs in the appropriate clinical setting.
